# Neighborhood socioeconomic deprivation, healthcare access, and 30-day mortality and readmission after sepsis or critical illness: findings from a nationwide study

**DOI:** 10.1186/s13054-023-04565-9

**Published:** 2023-07-15

**Authors:** Jay B. Lusk, Beau Blass, Hannah Mahoney, Molly N. Hoffman, Amy G. Clark, Jonathan Bae, Deepshikha C. Ashana, Christopher E. Cox, Bradley G. Hammill

**Affiliations:** 1 Duke University School of Medicine, Durham, NC USA; 2 Duke University Fuqua School of Business, Durham, NC USA; 3 Duke University Department of Population Health Sciences, 215 Morris St, Durham, NC 27701 USA; 4 Duke University Health System, Durham, NC USA; 5 Duke University Department of Medicine, Durham, NC USA

**Keywords:** Neighborhood deprivation, Sepsis, Socioeconomic disparities, Area deprivation index

## Abstract

**Background:**

To determine if neighborhood socioeconomic deprivation independently predicts 30-day mortality and readmission for patients with sepsis or critical illness after adjusting for individual poverty, demographics, comorbidity burden, access to healthcare, and characteristics of treating healthcare facilities.

**Methods:**

We performed a nationwide study of United States Medicare beneficiaries from 2017 to 2019. We identified hospitalized patients with severe sepsis and patients requiring prolonged mechanical ventilation, tracheostomy, or extracorporeal membrane oxygenation (ECMO) through Diagnosis Related Groups (DRGs). We estimated the association between neighborhood socioeconomic deprivation, measured by the Area Deprivation Index (ADI), and 30-day mortality and unplanned readmission using logistic regression models with restricted cubic splines. We sequentially adjusted for demographics, individual poverty, and medical comorbidities, access to healthcare services; and characteristics of treating healthcare facilities.

**Results:**

A total of 1,526,405 admissions were included in the mortality analysis and 1,354,548 were included in the readmission analysis. After full adjustment, 30-day mortality for patients was higher for those from most-deprived neighborhoods (ADI 100) compared to least deprived neighborhoods (ADI 1) for patients with severe sepsis (OR 1.35 95% [CI 1.29–1.42]) or with prolonged mechanical ventilation with or without sepsis (OR 1.42 [95% CI 1.31, 1.54]). This association was linear and dose dependent. However, neighborhood socioeconomic deprivation was not associated with 30-day unplanned readmission for patients with severe sepsis and was inversely associated with readmission for patients requiring prolonged mechanical ventilation with or without sepsis.

**Conclusions:**

A strong association between neighborhood socioeconomic deprivation and 30-day mortality for critically ill patients is not explained by differences in individual poverty, demographics, measured baseline medical risk, access to healthcare resources, or characteristics of treating hospitals.

**Supplementary Information:**

The online version contains supplementary material available at 10.1186/s13054-023-04565-9.

## Introduction

There is growing interest in understanding how neighborhood socioeconomic disadvantage contributes to health outcomes. Neighborhood disadvantage comprises many social and environmental factors which impact individual health, including economic stress, inadequate access to healthcare facilities, poor air quality, high-density housing, poorly maintained infrastructure, and lack of safe outdoor spaces [1–5]. In combination, these structural forces impact individual health in devastating ways. In fact, neighborhood disadvantage independently predicts certain poor health outcomes even after controlling for individual socioeconomic status, indicating that social and environmental barriers beyond the control of individual patients play a major role in driving health outcomes [1, 2, 6, 7]. In this way, neighborhood disadvantage represents an important area of consideration in work to promote health equity. Understanding the extent to which neighborhood characteristics are linked with health outcomes could provide targetable actions for public health improvement and guide the development of fair and equitable metrics for reimbursement and quality care improvement.

Neighborhood and environmental factors have previously been linked with some outcomes in critical care. For example, exposure to air pollution is associated with sepsis mortality [8]. Similarly, high neighborhood poverty is associated with increased incidence of bloodstream infections for patients receiving critical care, as well as higher sepsis-attributable mortality [9–11]. Prior research has also demonstrated significantly higher risk of readmission following sepsis hospitalization for patients from disadvantaged neighborhoods [12]. However, prior research has been restricted to institutional or geographically non-representative cohorts, and no study evaluating the association between neighborhood deprivation and outcomes for critically ill patients has been performed using large nationally representative datasets.

Furthermore, the relationship between neighborhood deprivation and outcomes may be confounded by other critical factors, including individual socioeconomic status, demographics, baseline medical comorbidities, severity of illness on initial presentation, access to both inpatient and outpatient healthcare resources, and hospital-level factors such as teaching status, size, ownership, and quality of care metrics. Therefore, in this study we aimed to test the hypothesis that neighborhood socioeconomic deprivation is associated with 30-day mortality and unplanned readmission, even after accounting for the effect of individual socioeconomic status and measured medical risk, healthcare access, and quality of treating healthcare facilities in a large, nationally representative sample.

## Methods

### Study population and data source

We utilized 100% of United States Medicare claims data from 2016 to 2019, obtained through a use agreement with the Centers for Medicare and Medicaid Services (CMS). Patients who were hospitalized between 2017 and 2019 were eligible for inclusion in the study; 2016 inpatient, outpatient, and carrier claim files were used for a 1-year prior comorbidity ascertainment. Participant demographics, enrollment periods, and mortality dates were obtained from the Medicare Beneficiary Summary Files (MBSF). We determined neighborhood socioeconomic status using the Area Deprivation Index (ADI), described below, which is measured at the census block group level. To link beneficiaries to census block groups, we utilized the ZIP + 4 code corresponding to beneficiary addresses. To identify patients admitted with common critical care conditions, we used diagnosis related groups (DRGs). DRGs identify the primary reason a patient was admitted to the hospital and are used to determine payments hospitals receive and to group similar diagnoses together into broader categories. For this study, we focused on two groups: patients admitted with septicemia or severe sepsis (DRG 870–872, subsequently referred to as severe sepsis), and patients who required extracorporeal membrane oxygenation (ECMO) or tracheostomy with invasive mechanical ventilation for longer than 96 h, or patients who had a respiratory system diagnosis other than sepsis requiring mechanical ventilatory support (DRG 003, 004, and 207–208, subsequently referred to as critical illness with or without sepsis). A breakdown of primary diagnoses by ICD-CM-10 codes are shown in Additional file 1: Tables S1 and S2. An illustration of the process through which patients with severe sepsis are assigned to Diagnosis Related Groups relating to either category is shown in Additional file 1: Table S3.

### Cohort creation

We created two cohorts, a mortality and a readmission cohort using inclusion/exclusion criteria similar to those established by CMS for outcomes assessment, detailed in brief below [13]. For the mortality cohort, inclusion criteria were age ≥ 65 years, 1 year of prior fee-for-service Medicare enrollment from admission date, and continuous enrollment in fee-for-service Medicare for at least 30-days following admission (or until death if patients died within this 30-day window). Patients transferred from another acute care hospital were excluded from the mortality cohort. Patients in the readmission cohort must have been discharged alive; patients who left against medical advice, who were transferred to another acute care hospital, and were not discharged on the same day of admission were excluded from the readmission cohort. For both cohorts, if a patient was hospitalized more than once in the study period, a single, random hospitalization per admission category was selected.

### Primary exposure

The primary exposure was neighborhood socioeconomic deprivation, measured through the validated Area Deprivation Index (ADI) [14–16]. In summary, the ADI uses socioeconomic indicators such as income, housing characteristics, unemployment, and educational attainment to estimate neighborhood socioeconomic deprivation at the census block group level [16].

### Statistical analysis

The characteristics of each study group were summarized using frequencies and percentages for categorical variables and means with standard deviations for continuous variables. For descriptive purposes, we used the Elixhauser Comorbidity Index to report overall comorbidity burden [17]. Comorbid conditions nearly universally present in any diagnosis group were excluded from adjustment: specifically, hypertension was excluded for the mechanical ventilation with or without sepsis group. We summarized the descriptive characteristics of the mortality and readmission cohorts by ADI groups and classified for these tables into high (ADI 1–15), middle (ADI 16–85), and low (ADI 86–100) neighborhood SES. However, to allow ADI to vary flexibly across its entire range of values for modeling, we included ADI as a continuous variable using a restricted cubic spline having four knots (at 5, 30, 70, and 95) [18]. All logistic regression models used generalized estimating equations to account for within-hospital clustering. We present results as Odds Ratios (ORs) with 95% confidence intervals (CI) and visualize the relationship between ADI outcomes using plots. For covariate adjustment**,** we utilized a sequential adjustment approach intended to uncover whether other factors associated with neighborhood socioeconomic deprivation explained the relationship between neighborhood deprivation and outcomes. Our first adjustment domain was for individual patient factors, which consisted of age, sex, race/ethnicity, Medicare-Medicaid dual-eligibility (an indicator of individual poverty) [19], and medical comorbidities, including end-stage renal disease status (per CMS methodology, as some patients qualify for Medicare due to end-stage renal disease status). We adjusted for the 29 comorbid medical conditions included in the Elixhauser mortality and readmission indices, ascertained in the prior year of claims [17, 20]. Our next adjustment domain was access to healthcare resources. Measures of healthcare access included number of primary care providers (PCPs) per capita, number of specialists per capita, and number of hospital beds per capita, which were obtained at the county level from the 2019 Area Health Resource File (AHRF), available from the Health Resources and Services Administration. Our final adjustment domain was admitting hospital characteristics. These characteristics were obtained from the 2018 American Hospital Association Annual Survey Database and included number of beds, ownership status (public vs. private), and teaching status. In a sensitivity analysis we additionally adjusted for additional indicators of sepsis severity, including in-hospital shock diagnosis, timing of mechanical ventilation, in-hospital use of hemodialysis (thereby replicating the approach of sepsis severity adjustment devised by Ford et al. [21]), indicators of hospital-level factors including performance on the Centers for Medicare and Medicaid services SEP-1 sepsis quality metric and hospital-wide readmission metric. Finally, we included additional pre-hospital healthcare utilization metrics such as admission from a skilled nursing facility and prior hospital discharge within 30 days. All analyses were conducted in SAS (SAS Institute, Cary, NC). This study was approved by the Duke University Institutional Review Board. Findings are reported according to the recommendations in the REporting of studies Conducted using Observational Routinely-collected health Data (RECORD) statement.

## Results

A total of 1,486,683 admissions were included in the mortality analysis and 1,311,373 were included in the readmission analysis. In the mortality analysis, 91.2% were in the severe sepsis group and 8.8% were in the mechanical ventilation with or without sepsis group. In the readmission analysis, 92.4% were in the severe sepsis group and 7.6% were in the mechanical ventilation with or without sepsis group.

Demographics and observed outcomes of the mortality and readmission cohorts by admission group are shown in Tables 1 and 2, respectively.

**Table 1:** Demographics, 30-day outcomes, access to healthcare resources, and characteristics of admitting healthcare facilities, stratified by admission groups within the mortality cohort

Variable	Severe sepsis (N = 1,343, 414)	Mechanically ventilated with or without sepsis (N = 143,269)
Main outcome		
Observed mortality	325,704 (24.2%)	56,116 (39.2%)
Demographics		
Age (years), mean (SD)	79.5 (8.6)	76.3 (7.6)
Legal sex, female	715,349 (53.2%)	72,929 (50.9%)
Dual medicare/medicaid eligible	386,882 (28.8%)	45,478 (31.7%)
Race/ethnicity		
Asian	28,033 (2.1%)	3114 (2.2%)
Black	117,011 (8.7%)	21,336 (14.9%)
Hispanic	26,287 (2.0%)	2849 (2.0%)
Other/Unknown	38,661 (2.9%)	4463 (3.1%)
White	1,133,422 (84.4%)	111,507 (77.8%)
Medical history and in-hospital indicators		
Elixhauser mortality index, mean (SD)	25.3 (15.5)	30.6 (14.8)
Admission from SNF	90,412 (6.7)	7605 (5.3)
Prior discharge within 30 days	250,776 (18.7)	36,006 (25.1)
Mechanical ventilation timing		
None	1,218,495 (90.7)	2584 (1.8)
Early	104,708 (7.8)	106,870 (74.6)
Late	20,211 (1.5)	33,815 (23.6)
In-hospital shock diagnosis	256,032 (19.1)	26,173 (18.3)
In-hospital dialysis procedure	39,592 (2.9)	10,146 (7.1)
In-hospital ICU utilization	619,632 (46.1)	126,975 (88.6)
Length of stay, days, mean (SD)	5.8 (5.0)	13.1 (15.5)
Discharge to post-acute care (SNF, rehab, etc.)	419,104 (31.2)	56,703 (39.6)
Regional information		
Rural area	252,728 (18.8%)	24,625 (17.2%)
Primary care providers per 100,000 persons, mean (SD)	74.6 (32.2)	73.7 (31.7)
Total specialists per 100,000 persons, mean (SD)	221.2 (179.0)	224.6 (179.4)
Total pulmonary disease specialists per 1,000,000 persons, mean (SD)	39.5 (38.2)	40.6 (38.3)
Hospital beds per 10,000 persons, mean (SD)	28.4 (21.6)	29.4 (21.7)
Distance to the closest hospital, miles, mean (SD)	4.1 (5.2)	4.0 (5.1)
Hospital information		
Number of beds, mean (SD)	353.8 (320.6)	420.6 (357.0)
Ownership, public	135,085 (10.1%)	16,036 (11.2%)
Teaching hospital	186,660 (13.9%)	27,956 (19.5%)
CMS metric, SEP-1, mean (SD)	57.7 (15.5)	56.3 (15.8)
CMS metric, hospital-wide readmission, mean (SD)	15.3 (0.9)	15.4 (0.9)

*SD* standard deviation

**Table 2:** Demographics, 30-day outcomes, access to healthcare resources, and characteristics of admitting healthcare facilities, stratified by admission groups within the readmission cohort

Variable	Severe Sepsis (N = 1,203,345)	Mechanically ventilated with or without sepsis (N = 108,028)
Main outcome		
Observed readmission	199,348 (16.6%)	22,818 (21.1%)
Demographics		
Age (years), mean (SD)	79.3 (8.5)	75.6 (7.4)
Legal sex, female	641,801 (53.3%)	56,110 (51.9%)
Dual medicare/medicaid eligible	344,374 (28.6%)	36,874 (34.1%)
Race/ethnicity		
Asian	23,916 (2.0%)	2079 (1.9%)
Black	100,528 (8.4%)	16,234 (15.0%)
Hispanic	23,038 (1.9%)	2,056 (1.9%)
Other/unknown	34,927 (2.9%)	3379 (3.1%)
White	1,020,936 (84.8%)	84,280 (78.0%)
Medical history and in-hospital indicators		
Elixhauser readmission index, mean (SD)	55.7 (27.1)	65.8 (25.8)
Admission from SNF	71,212 (5.9)	4866 (4.5)
Prior discharge within 30 days	190,069 (15.8)	24,855 (23.0)
Mechanical ventilation timing		
None	1,136,813 (94.5)	2253 (2.1)
Early	58,293 (4.8)	83,355 (77.2)
Late	8239 (0.7)	22,420 (20.8)
In-hospital shock diagnosis	175,040 (14.5)	15,918 (14.7)
In-hospital dialysis procedure	31,804 (2.6)	6965 (6.4)
In-hospital ICU utilization	523,296 (43.5)	98,493 (91.2)
Length of stay, days, mean (SD)	6.0 (4.8)	14.6 (14.4)
Discharge to post-acute care (SNF, rehab, etc.)	445,860 (37.1)	65,414 (60.6)
Regional information		
Rural area	239,184 (19.9%)	23,023 (21.3%)
Primary care providers per 100,000 persons, mean (SD)	74.0 (32.2)	71.6 (31.8)
Total specialists per 100,000 persons, mean (SD)	217.4 (178.3)	212.6 (177.9)
Total pulmonary disease specialists per 1,000,000 persons, mean (SD)	38.7 (38.1)	38.3 (38.1)
Hospital beds per 10,000 persons, mean (SD)	28.3 (21.9)	29.2 (22.7)
Distance to the closest hospital, miles, mean (SD)	4.1 (5.2)	4.1 (5.3)
Hospital information		
Number of beds, mean (SD)	361.5 (323.0)	450.8 (361.7)
Ownership, public	118,370 (9.8%)	12,120 (11.2%)
Teaching hospital	176,284 (14.6%)	25,440 (23.5%)
CMS metric, SEP-1, mean (SD)	57.5 (15.5)	55.6 (15.9)
CMS metric, hospital-wide readmission, mean (SD)	15.3 (0.9)	15.4 (0.9)

*SD* standard deviation

Demographic characteristics, access to healthcare resources, and observed outcomes, stratified by neighborhood SES are shown in Table 3. Generally, patients in the low neighborhood SES group were younger, more likely to be female, less likely to be white, and more often dually eligible for Medicare-Medicaid. Observed mortality rates were higher for low neighborhood SES compared to high and middle SES. These same demographic trends were observed for the readmission cohort, for which patients in the low neighborhood SES group also experienced higher rates of readmission overall (Table 3).

**Table 3:** Demographics, 30-day outcomes, access to healthcare resources, and characteristics of admitting healthcare facilities, stratified by neighborhood socioeconomic status group within the mortality and readmission cohorts

Variable	High neighborhood SES/ADI 1–15 (N = 215,199)	Middle neighborhood SES/ADI 16–85 (N = 1,090,403)	Low neighborhood SES/ADI 86–100 (N = 181,081)
Mortality cohort			
Main outcome			
Observed mortality	54,860 (25.5%)	277,542 (25.5%)	49,418 (27.3%)
Demographics			
Age (years), mean (SD)	81.3 (8.8)	79.0 (8.5)	77.8 (8.4)
Legal sex, female	111,014 (51.6%)	577,460 (53.0%)	99,804 (55.1%)
Race/ethnicity			
Asian	14,819 (6.9%)	15,514 (1.4%)	814 (0.4%)
Black	12,059 (5.6%)	87,927 (8.1%)	38,361 (21.2%)
Hispanic	4522 (2.1%)	20,296 (1.9%)	4318 (2.4%)
Other/Unknown	11,310 (5.3%)	27,116 (2.5%)	4,698 (2.6%)
White	172,489 (80.2%)	939,550 (86.2%)	132,890 (73.4%)
Dual medicare/medicaid eligible	58,308 (27.1%)	295,706 (27.1%)	78,346 (43.3%)
Medical history and in-hospital indicators			
Elixhauser mortality index, mean (SD)	26.9 (15.9)	25.5 (15.4)	26.1 (15.3)
Admission from SNF	18,651 (8.7)	69,740 (6.4)	9626 (5.3)
Prior discharge within 30 days	40,979 (19.0)	208,522 (19.1)	37,281 (20.6)
Mechanical ventilation timing			
None	178,870 (83.1)	899,694 (82.5)	142,515 (78.7)
Early	28,322 (13.2)	152,242 (14.0)	31,014 (17.1)
Late	8007 (3.7)	38,467 (3.5)	7552 (4.2)
In-hospital shock diagnosis	43,613 (20.3)	202,948 (18.6)	35,644 (19.7)
In-hospital dialysis procedure	7087 (3.3)	34,844 (3.2)	7807 (4.3)
In-hospital ICU utilization	100,522 (46.7)	549,467 (50.4)	96,618 (53.4)
Length of stay, days, mean (SD)	7.1 (8.6)	6.4 (6.8)	6.6 (7.1)
Discharge to post-acute care (SNF, rehab, etc.)	68,189 (31.7)	349,375 (32.0)	58,243 (32.2)
Regional information			
Rural area	2,505 (1.2%)	200,435 (18.4%)	74,413 (41.1%)
Primary care providers per 100,000 persons, mean (SD)	95.3 (29.0)	72.6 (31.1)	61.1 (30.6)
Total specialists per 100,000 persons, mean (SD)	343.9 (198.6)	208.4 (167.7)	155.4 (154.4)
Total pulmonary disease specialists per 1,000,000 persons, mean (SD)	59.2 (38.9)	37.5 (37.1)	28.9 (35.8)
Hospital beds per 10,000 persons, mean (SD)	26.5 (14.0)	28.1 (21.2)	33.4 (29.7)
Distance to the closest hospital, miles, mean (SD)	2.6 (2.9)	4.2 (5.2)	4.7 (6.7)
Hospital information			
Number of beds, mean (SD)	420.0 (361.3)	353.0 (320.6)	333.1 (295.0)
Ownership, public	18,938 (8.8%)	106,712 (9.8%)	25,471 (14.1%)
Teaching hospital	54,290 (25.2%)	140,112 (12.8%)	20,214 (11.2%)
CMS metric, SEP-1, mean (SD)	60.0 (14.9)	57.3 (15.5)	56.5 (15.9)
CMS metric, Hospital-wide readmission, mean (SD)	15.3 (1.0)	15.3 (0.9)	15.5 (0.9)
Readmission cohort			
Main outcome			
Observed readmission	30,949 (16.9%)	162,117 (16.8%)	29,100 (18.0%)
Demographics			
Age (years), mean (SD)	81.1 (8.7)	78.8 (8.4)	77.6 (8.3)
Legal sex, female	94,950 (51.8%)	513,223 (53.1%)	89,738 (55.4%)
Race/ethnicity			
Asian	12,222 (6.7%)	13,074 (1.4%)	699 (0.4%)
Black	9837 (5.4%)	74,384 (7.7%)	32,541 (20.1%)
Hispanic	3841 (2.1%)	17,517 (1.8%)	3736 (2.3%)
Other/Unknown	9562 (5.2%)	24,210 (2.5%)	4534 (2.8%)
White	147,948 (80.7%)	836,812 (86.6%)	120,456 (74.4%)
Dual medicare/medicaid eligible	48,994 (26.7%)	261,920 (27.1%)	70,334 (43.4%)
Medical history and in-hospital indicators			
Elixhauser readmission index, mean (SD)	56.2 (27.6)	56.1 (27.1)	59.1 (27.0)
Admission from SNF	14,407 (7.9)	54,247 (5.6)	7424 (4.6)
Prior discharge within 30 days	29,043 (15.8)	157,216 (16.3)	28,665 (17.7)
Mechanical ventilation timing			
None	162,990 (88.9)	841,264 (87.1)	134,812 (83.2)
Early	16,424 (9.0)	102,525 (10.6)	22,699 (14.0)
Late	3996 (2.2)	22,208 (2.3)	4455 (2.8)
In-hospital shock diagnosis	26,886 (14.7)	138,967 (14.4)	25,105 (15.5)
In-hospital dialysis procedure	4939 (2.7)	27,429 (2.8)	6,401 (4.0)
In-hospital ICU utilization	78,661 (42.9)	459,942 (47.6)	83,186 (51.4)
Length of stay, days, mean (SD)	7.1 (7.7)	6.6 (6.4)	6.9 (6.3)
Discharge to post-acute care (SNF, rehab, etc.)	71,076 (38.8)	375,390 (38.9)	64,808 (40.0)
Regional information			
Rural area	2,264 (1.2%)	189,860 (19.7%)	70,083 (43.3%)
Primary care providers per 100,000 persons, mean (SD)	95.2 (28.9)	72.1 (31.1)	60.1 (30.6)
Total specialists per 100,000 persons, mean (SD)	342.1 (197.3)	204.6 (167.3)	149.5 (152.0)
Total pulmonary disease specialists per 1,000,000 persons, mean (SD)	58.8 (38.8)	36.7 (37.1)	27.7 (35.3)
Hospital beds per 10,000 persons, mean (SD)	26.4 (14.0)	28.0 (21.4)	33.0 (30.4)
Distance to the closest hospital, miles, mean (SD)	2.7 (3.0)	4.3 (5.2)	4.7 (6.8)
Hospital information			
Number of beds, mean (SD)	423.0 (361.7)	362.1 (323.6)	347.7 (300.7)
Ownership, public	15,823 (8.6%)	92,587 (9.6%)	22,080 (13.6%)
Teaching hospital	47,084 (25.7%)	134,468 (13.9%)	20,172 (12.5%)
CMS metric, SEP-1, mean (SD)	60.0 (15.0)	57.1 (15.5)	56.3 (15.9)
CMS metric, hospital-wide readmission, mean (SD)	15.3 (0.9)	15.3 (0.9)	15.4 (0.9)

*ADI* area deprivation index, *SES* socioeconomic status, *SD* standard deviation. Differences between groups were significant for all variables at p < .001

Unadjusted and sequentially adjusted estimates of the association between neighborhood deprivation and 30-day mortality are shown in Table 4, stratified by admission group.

**Table 4:** Regression-estimated effects of neighborhood socioeconomic status on odds of 30-day mortality and readmission

Group	ADI percentile	Model 1 (unadjusted) OR (95% CI)	Model 2 (+patient characteristics) OR (95% CI)	Model 3 (+healthcare access) OR (95% CI)	Model 4 (+hospital characteristics) OR (95% CI)	Model 5 (+additional sensitivity adjustment) OR (95% CI)
Severe sepsis (Outcome of 30-day mortality)	1	1.00 (Ref)	1.00 (Ref)	1.00 (Ref)	1.00 (Ref)	1.00 (Ref)
	15	0.98 (0.96, 0.99)	1.03 (1.02, 1.04)	1.03 (1.02, 1.04)	1.06 (1.04, 1.09)	1.08 (1.06, 1.10)
	50	0.98 (0.95, 1.00)	1.11 (1.09, 1.13)	1.11 (1.09, 1.14)	1.20 (1.15, 1.25)	1.24 (1.19, 1.29)
	85	1.05 (1.02, 1.07)	1.19 (1.17, 1.22)	1.20 (1.17, 1.22)	1.30 (1.25, 1.36)	1.33 (1.28, 1.38)
	100	1.08 (1.05, 1.11)	1.23 (1.19, 1.26)	1.23 (1.20, 1.27)	1.35 (1.29, 1.42)	1.36 (1.30, 1.43)
Mechanically ventilated with or without sepsis (Outcome of 30-day mortality)	1	1.00 (Ref)	1.00 (Ref)	1.00 (Ref)	1.00 (Ref)	1.00 (Ref)
	15	1.01 (0.98, 1.05)	1.10 (1.06, 1.14)	1.10 (1.06, 1.14)	1.12 (1.08, 1.16)	1.14 (1.10, 1.19)
	50	1.02 (0.97, 1.08)	1.24 (1.17, 1.32)	1.25 (1.18, 1.33)	1.31 (1.22, 1.40)	1.37 (1.27, 1.47)
	85	0.96 (0.92, 1.02)	1.29 (1.22, 1.36)	1.31 (1.24, 1.39)	1.37 (1.28, 1.46)	1.44 (1.34, 1.54)
	100	0.92 (0.87, 0.99)	1.34 (1.25, 1.43)	1.36 (1.26, 1.46)	1.42 (1.31, 1.54)	1.49 (1.37, 1.62)
Severe Sepsis (Outcome of 30-day readmission)	1	1.00 (Ref)	1.00 (Ref)	1.00 (Ref)	1.00 (Ref)	1.00 (Ref)
	15	1.02 (1.00, 1.04)	0.98 (0.97, 1.00)	0.99 (0.97, 1.00)	0.98 (0.96, 1.00)	0.97 (0.96, 0.99)
	50	1.06 (1.03, 1.09)	0.97 (0.95, 1.00)	0.98 (0.95, 1.00)	0.96 (0.94, 0.99)	0.95 (0.93, 0.98)
	85	1.13 (1.10, 1.16)	0.99 (0.97, 1.01)	0.99 (0.97, 1.02)	0.98 (0.95, 1.01)	0.97 (0.94, 0.99)
	100	1.18 (1.15, 1.22)	0.99 (0.96, 1.03)	1.00 (0.97, 1.03)	0.99 (0.95, 1.02)	0.96 (0.93, 0.99)
Mechanically ventilated with or without sepsis (Outcome of 30-day readmission)	1	1.00 (Ref)	1.00 (Ref)	1.00 (Ref)	1.00 (Ref)	1.00 (Ref)
	15	0.99 (0.95, 1.04)	0.97 (0.93, 1.02)	0.98 (0.94, 1.03)	0.97 (0.93, 1.02)	0.98 (0.93, 1.03)
	50	0.96 (0.89, 1.03)	0.91 (0.84, 0.99)	0.94 (0.87, 1.02)	0.90 (0.83, 0.99)	0.92 (0.85, 1.01)
	85	0.97 (0.90, 1.04)	0.88 (0.82, 0.94)	0.91 (0.85, 0.99)	0.86 (0.79, 0.94)	0.88 (0.81, 0.96)
	100	0.99 (0.91, 1.09)	0.87 (0.80, 0.96)	0.91 (0.83, 1.00)	0.86 (0.77, 0.95)	0.87 (0.78, 0.96)

*ADI* area deprivation index, *CI* confidence interval, *OR* odds ratio

Model 1 covariate included ADI restricted cubic spline terms only. Model 2 added covariates for age, sex, race/ethnicity, year of admission, end-stage renal disease status, and comorbid conditions. Model 3 added covariates for residence in a rural area, number of primary care providers per 100,000 persons, total number of specialists per 100,000 persons, hospital beds per 10,000 persons, and distance to the nearest hospital in miles. Model 4 added covariates for number of beds of admitting hospital, teaching status of admitting hospital, and public vs. private ownership of admitting hospital. Model 5 was a sensitivity analysis additionally adjusting for: admission from a skilled nursing facility, prior hospital discharge within 30 days, timing of mechanical ventilation (none vs. early vs. late), in-hospital shock diagnosis, in-hospital dialysis, in-hospital ICU utilization, hospital-level CMS SEP-1 scores, hospital-level CMS hospital-wide readmission scores. In the readmission analysis, Model 5 also included length of stay and disposition (post-acute care versus home)

Model 1 covariate included ADI restricted cubic spline terms only. Model 2 added covariates for age, sex, race/ethnicity, year of admission, end-stage renal disease status, and comorbid conditions. Model 3 added covariates for residence in a rural area, number of primary care providers per 100,000 persons, total number of specialists per 100,000 persons, hospital beds per 10,000 persons, and distance to the nearest hospital in miles. Model 4 added covariates for number of beds of admitting hospital, teaching status of admitting hospital, and public vs. private ownership of admitting hospital. Model 5 was a sensitivity analysis additionally adjusting for: admission from a skilled nursing facility, prior hospital discharge within 30 days, timing of mechanical ventilation (none vs. early vs. late), in-hospital shock diagnosis, in-hospital dialysis, in-hospital ICU utilization, hospital-level CMS SEP-1 scores, hospital-level CMS hospital-wide readmission scores. In the readmission analysis, Model 5 also included length of stay and disposition (post-acute care versus home).

Before adjustment, a modest relationship between neighborhood SES and 30-day mortality was seen only for the severe sepsis group (OR 1.08 [95% CI 1.05, 1.11] for patients from most-deprived neighborhoods vs those from least-deprived neighborhoods). After adjusting for patient characteristics, a strong, dose-dependent relationship between area deprivation and mortality was observed for both disease groups. This relationship was accentuated after adjusting for metrics of access to healthcare and bolstered again after subsequent adjustment for characteristics of the treating hospitals. Put simply, the strongest relationship between neighborhood SES and 30-day mortality was observed in the fully adjusted model (aOR 1.35 [95% CI 1.29–1.42] for the severe sepsis group and aOR 1.42 [95% 1.31–1.54] for the mechanically ventilated without sepsis group.

**Fig. 1:**
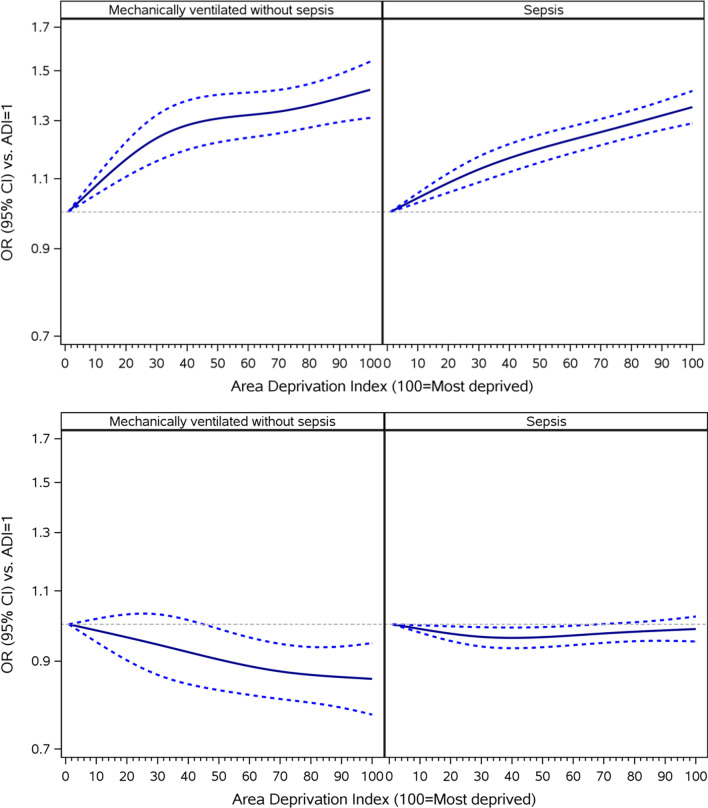
Adjusted* associations between neighborhood-level socioeconomic status and 30-day mortality (top) and readmission (bottom) for patients admitted with critical illness. *Adjustment covariates included age, sex, Medicare-Medicaid dual eligibility status, end-stage renal disease status, discharge year, the Elixhauser comorbiditity conditions, residence in a rural area, number of primary care providers per 100,000 persons, total number of specialists per 100,000 persons, hospital beds per 10,000 persons, distance to the nearest hospital in miles, number of beds of admitting hospital, teaching status of admitting hospital, and public vs. private ownership of admitting hospital

Unadjusted and sequentially adjusted estimates of the effect of neighborhood SES on 30-day unplanned readmission are shown in Table 4, stratified by admission group. Prior to adjustment, there was a modest relationship between neighborhood SES and readmission for the severe sepsis group (OR 1.18 [95% CI 1.55–1.22]). However, this association was lost after adjusting for patient characteristics. Subsequent adjustments for access to healthcare and hospital characteristics elucidated no new additional relationships for the severe sepsis group (aOR 0.99 [95% CI 0.95–1.02]). By contrast, a weak negative relationship was observed between neighborhood SES and 30-day readmission for the mechanically ventilated without sepsis group (fully adjusted aOR 0.86 [95% CI 0.77, 0.95]). A more precise characterization of the relationship between neighborhood deprivation and mortality or readmission from the fully adjusted model for both disease groups is depicted in Fig. 1.

An additional sensitivity analysis which adjusted for additional indicators of patient-level risk as well as additional indicators of healthcare utilization and hospital level performance measures did not result in substantially different estimates of the association between neighborhood socioeconomic status and 30-day mortality and readmission (Tables 3 and 4). Furthermore, a sensitivity analysis comparing our primary analysis method, which estimated average within-hospital effects of the association between neighborhood socioeconomic deprivation and mortality (utilizing generalized estimating equations) versus unconditional on-hospital estimates that do not adjust for across-hospital effects showed more extreme estimates of the association between neighborhood socioeconomic deprivation and mortality, suggesting non-random clustering of similar patients within hospitals by performance (Additional file 1: Table S4).

## Discussion

Our study demonstrates that neighborhood socioeconomic deprivation is strongly associated with 30-day mortality for critically ill patients even after adjustment for patient demographics, medical comorbidities, individual poverty, access to healthcare resources, and characteristics of treating healthcare facilities. By contrast, our study demonstrates that neighborhood socioeconomic deprivation is not strongly associated or is inversely associated with 30-day readmission for patients with critical illness.

Our study advances the literature on the association between neighborhood characteristics and sepsis mortality. Prior studies evaluating variation in sepsis mortality on the community level or studying patient-level outcomes in smaller, geographically restricted cohorts have suggested that neighborhood deprivation is associated with greater mortality rates for patients with sepsis [11, 22]. By contrast, a prospective, observational cohort study in France suggested that there was no association between socioeconomic deprivation and 3-month mortality [23]. Our study demonstrates that, among Medicare beneficiaries in the United States, there is a strong association between neighborhood SES and 30-day mortality.

Furthermore, our study evaluates whether access to healthcare resources and characteristics of treating healthcare facilities explain the relationship between neighborhood deprivation and mortality, as has been posited by prior studies [22]. We showed that the association between neighborhood deprivation and mortality persisted after adjustment for both access to healthcare and characteristics of treating healthcare facilities, demonstrating that the effect of neighborhood deprivation is not explained by insufficient healthcare access or greater propensity for some patients to be treated at certain hospitals.

While there was no adjusted association between neighborhood socioeconomic status and 30-day readmission for patients with severe sepsis, there was an inverse association between neighborhood deprivation and 30-day readmission for patients who were mechanically ventilated without severe sepsis. There are several possibilities for why we observed this inverse relationship. First, it is possible that patients with acute respiratory collapse (the most common individual diagnosis in this group) from low SES neighborhoods were more likely to have more severe pathology at baseline, leading to greater mortality rates (which prevents readmission); patients from low SES neighborhoods who survived the incident admission might be systematically healthier in ways that are not captured by claims data. Alternatively, there may be differential patterns of post-hospitalization care that resulted in greater readmission rates for patients from high SES neighborhoods. Future research should carefully evaluate patterns of post-discharge care utilization to shed additional light on this finding.

The magnitude of the effect of neighborhood socioeconomic status on 30-day mortality was large. Patients in the 85th percentile of neighborhood deprivation with severe sepsis had 30% higher rates of 30-day mortality, and mechanically ventilated patients without sepsis had 37% higher rates of 30-day mortality, compared to patients from least-deprived neighborhoods. These effects are particularly impressive in the context of established interventions to lower sepsis mortality. For example, prior research has shown that each increased hour from patient registration to antibiotic administration for patients with sepsis is associated with a 9% greater odds of in-hospital mortality: therefore if neighborhood socioeconomic status had a direct causal effect on mortality, ameliorating the harmful effect of neighborhood deprivation could provide a similar protective benefit as speeding the administration of antibiotics by 3 h [24]. Future studies to explicitly evaluate this causal pathway would be merited.

Our study has limitations. First, this was a retrospective analysis of Medicare claims data: as such, our findings may not be generalizable to patients under the age of 65 or patients insured through other mechanisms. Furthermore, claims data may lack granular clinical information and may fail to capture the extent of baseline medical risk that may vary among patients from neighborhoods of differing socioeconomic status. Additionally, our cohort identification strategy is limited by its use of diagnosis related groups instead of international classification of diseases (ICD) codes. While diagnosis related groups effectively identify groups of reasons that patients are hospitalized, they are less granular than individualized ICD codes. We were also unable to comprehensively adjust for patients’ levels of pre-hospitalization healthcare utilization and it is therefore possible that patients from neighborhoods of different socioeconomic statuses have differing levels of healthcare utilization, which could bias our results. However, our results were robust to adjustment for a broad range of healthcare utilization and access variables as well as variables indicating hospital level performance.

## Conclusions

Strategies to improve mortality among patients from socioeconomically deprived neighborhoods are urgently needed to improve health equity and survival for critically ill patients. Furthermore, our study has major implications for the evaluation of hospital performance, as applying penalties for 30-day mortality may inadvertently disadvantage safety net hospitals that care for a highly socioeconomically disadvantaged population.

## Supplementary Information

**Additional file 1**. **Table S1**: Primary ICD-10-CM Diagnosis Codes for Patients in the Readmission Cohort. **Table S2**: Primary ICD-10-CM Diagnosis Codes for the Mortality Cohort. **Table S3**: In-hospital factors determining assignment to DRG 003 versus DRG 004. **Table S4**: Results of unconditional-on-hospital models (i.e. those not utilizing generalized estimating equations) for the mortality and readmission cohorts.

## Data Availability

The datasets used and/or analyzed during the current study are available from the Centers for Medicare and Medicaid Services under an approved data use agreement.

## Abbreviations

ADI: Area deprivation Index
CMS: Centers for Medicare and Medicaid Services
SES: Socioeconomic status
OR: Odds ratio
AHRF: Area Health Resources File
MBSF: Medicare Beneficiary Summary Files
