# Supplementary material for: Neighborhood socioeconomic deprivation, healthcare access, and 30-day mortality and readmission after sepsis or critical illness: findings from a nationwide study
Source: Crit Care. 2023 Jul 15;27:287. doi: 10.1186/s13054-023-04565-9 (PMC10349422; doi:10.1186/s13054-023-04565-9)
Supplement: Supplementary file 1 — Additional file 1. Table S1: Primary ICD-10-CM Diagnosis Codes for Patients in the Readmission Cohort. Table S2: Primary ICD-10-CM Diagnosis Codes for the Mortality Cohort. Table S3: In-hospital factors determining assignment to DRG 003 versus DRG 004. Table S4: Results of unconditional-on-hospital models (i.e. those not utilizing generalized estimating equations) for the mortality and readmission cohorts. [file 13054_2023_4565_MOESM1_ESM.docx]

**Table S1. Primary ICD-10-CM Diagnosis Codes for Patients in the Readmission Cohort**

| *Study Group* | *ICD-10-CM Code* | *Description* | *Count* | *Percent* |
| --- | --- | --- | --- | --- |
| *Severe Sepsis* | A41 | Other sepsis | 1,151,033 | 95.7% |
|  | A40 | Streptococcal sepsis | 34,699 | 2.9% |
|  | R78 | Findings of drugs and other substances, not normally found in blood | 12,017 | 1.0% |
|  | R57 | Shock, not elsewhere classified | 1,686 | 0.1% |
|  | B37 | Candidiasis | 1,428 | 0.1% |
|  | R65 | Symptoms and signs specifically associated with systemic inflammation and infection | 1,242 | 0.1% |
|  | A02 | Other salmonella infections | 803 | 0.1% |
| *Mechanically ventilated with or without sepsis* | J96 | Respiratory failure, not elsewhere classified | 52,836 | 48.9% |
|  | A41 | Other sepsis | 7,590 | 7.0% |
|  | J44 | Other chronic obstructive pulmonary disease | 6,591 | 6.1% |
|  | J69 | Pneumonitis due to solids and liquids | 5,470 | 5.1% |
|  | J18 | Pneumonia, unspecified organism | 3,998 | 3.7% |
|  | J15 | Bacterial pneumonia, not elsewhere classified | 1,961 | 1.8% |
|  | S06 | Intracranial injury | 1,665 | 1.5% |
|  | I63 | Cerebral infarction | 1,652 | 1.5% |
|  | I26 | Pulmonary embolism | 1,358 | 1.3% |
|  | I61 | Nontraumatic intracerebral hemorrhage | 1,215 | 1.1% |
|  | J95 | Intraoperative and postprocedural complications and disorders of respiratory system, not elsewhere classified | 1,187 | 1.1% |
|  | I21 | Acute myocardial infarction | 1,136 | 1.1% |
|  | J10 | Influenza due to other identified influenza virus | 936 | 0.9% |
|  | C34 | Malignant neoplasm of bronchus and lung | 786 | 0.7% |
|  | I25 | Chronic ischemic heart disease | 698 | 0.6% |
|  | S22 | Fracture of rib(s), sternum and thoracic spine | 658 | 0.6% |
|  | S27 | Injury of other and unspecified intrathoracic organs | 592 | 0.5% |
|  | I71 | Aortic aneurysm and dissection | 579 | 0.5% |
|  | T17 | Foreign body in respiratory tract | 535 | 0.5% |
|  | I60 | Nontraumatic subarachnoid hemorrhage | 504 | 0.5% |

**Table S2. Primary ICD-10-CM Diagnosis Codes for Patients in the Mortality Cohort**

| *Study Group* | *ICD-10-CM Code* | *Description* | *Count* | *Percent* |
| --- | --- | --- | --- | --- |
| *Severe Sepsis* | A41 | Other sepsis | 1,288,300 | 95.9% |
|  | A40 | Streptococcal sepsis | 36,780 | 2.7% |
|  | R78 | Findings of drugs and other substances, not normally found in blood | 12,005 | 0.9% |
|  | R57 | Shock, not elsewhere classified | 1,917 | 0.1% |
|  | B37 | Candidiasis | 1,665 | 0.1% |
|  | R65 | Symptoms and signs specifically associated with systemic inflammation and infection | 1,443 | 0.1% |
|  | A02 | Other salmonella infections | 814 | 0.1% |
| *Mechanically ventilated with or without sepsis* | J96 | Respiratory failure, not elsewhere classified | 70,347 | 49.1% |
|  | J44 | Other chronic obstructive pulmonary disease | 8,921 | 6.2% |
|  | A41 | Other sepsis | 8,872 | 6.2% |
|  | J69 | Pneumonitis due to solids and liquids | 8,236 | 5.7% |
|  | J18 | Pneumonia, unspecified organism | 7,239 | 5.1% |
|  | I26 | Pulmonary embolism | 3,229 | 2.3% |
|  | J15 | Bacterial pneumonia, not elsewhere classified | 2,800 | 2.0% |
|  | S06 | Intracranial injury | 1,617 | 1.1% |
|  | C34 | Malignant neoplasm of bronchus and lung | 1,615 | 1.1% |
|  | I63 | Cerebral infarction | 1,420 | 1.0% |
|  | J10 | Influenza due to other identified influenza virus | 1,341 | 0.9% |
|  | J95 | Intraoperative and postprocedural complications and disorders of respiratory system, not elsewhere classified | 1,294 | 0.9% |
|  | I21 | Acute myocardial infarction | 1,230 | 0.9% |
|  | T17 | Foreign body in respiratory tract | 1,029 | 0.7% |
|  | I61 | Nontraumatic intracerebral hemorrhage | 964 | 0.7% |
|  | I25 | Chronic ischemic heart disease | 934 | 0.7% |
|  | S22 | Fracture of rib(s), sternum and thoracic spine | 863 | 0.6% |
|  | S27 | Injury of other and unspecified intrathoracic organs | 766 | 0.5% |
|  | J84 | Other interstitial pulmonary diseases | 646 | 0.5% |
|  | I35 | Nonrheumatic aortic valve disorders | 606 | 0.4% |
|  | I71 | Aortic aneurysm and dissection | 604 | 0.4% |
|  | I13 | Hypertensive heart and chronic kidney disease | 510 | 0.4% |

**Table S3. In-hospital factors determining assignment to DRG 003 versus DRG 004**

| **ECMO** | **Tracheostomy** | **Mechanical Ventilation >96 hours** | **Primary Diagnosis Excluding Face, Mouth, Neck** | **Major O.R. Procedure** | **DRG Assigned** |
| --- | --- | --- | --- | --- | --- |
| Yes | n/a | n/a | n/a | n/a | 003 |
| No | Yes | Yes |  | Yes | 003 |
| No | Yes |  | Yes | Yes | 003 |
| No | Yes | Yes |  | No | 004 |
| No | Yes |  | Yes | No | 004 |

**Table S4. Results of unconditional-on-hospital models (i.e., those not utilizing generalized estimating equations) for the mortality and readmission cohorts**

***Mortality cohort***

| **Group** | **ADI Percentile** | **Model 1**  **(Unadjusted)**  **OR (95% CI)** | **Model 2**  **(+Patient characteristics)**  **OR (95% CI)** |
| --- | --- | --- | --- |
| Severe Sepsis | 1 | 1.00 (Ref) | 1.00 (Ref) |
|  | 15 | 0.97 (0.96, 0.98) | 1.07 (1.06, 1.08) |
|  | 50 | 0.95 (0.94, 0.97) | 1.22 (1.20, 1.24) |
|  | 85 | 1.04 (1.03, 1.05) | 1.33 (1.31, 1.36) |
|  | 100 | 1.10 (1.08, 1.13) | 1.39 (1.35, 1.42) |
|  |  |  |  |
| Mechanically ventilated with or without sepsis | 1 | 1.00 (Ref) | 1.00 (Ref) |
|  | 15 | 1.06 (1.03, 1.09) | 1.15 (1.11, 1.18) |
|  | 50 | 1.14 (1.08, 1.19) | 1.37 (1.30, 1.44) |
|  | 85 | 1.07 (1.02, 1.11) | 1.43 (1.36, 1.49) |
|  | 100 | 1.02 (0.97, 1.08) | 1.48 (1.39, 1.58) |

***Readmission cohort***

| **Group** | **ADI Percentile** | **Model 1**  **(Unadjusted)**  **OR (95% CI)** | **Model 2**  **(+Patient characteristics)**  **OR (95% CI)** |
| --- | --- | --- | --- |
| Severe Sepsis | 1 | 1.00 (Ref) | 1.00 (Ref) |
|  | 15 | 0.99 (0.97, 1.00) | 0.97 (0.96, 0.98) |
|  | 50 | 0.98 (0.96, 1.00) | 0.95 (0.93, 0.97) |
|  | 85 | 1.05 (1.03, 1.07) | 0.97 (0.95, 0.99) |
|  | 100 | 1.11 (1.08, 1.14) | 0.97 (0.95, 1.00) |
|  |  |  |  |
| Mechanically ventilated with or without sepsis | 1 | 1.00 (Ref) | 1.00 (Ref) |
|  | 15 | 0.96 (0.92, 1.00) | 0.95 (0.91, 0.99) |
|  | 50 | 0.87 (0.82, 0.93) | 0.86 (0.80, 0.92) |
|  | 85 | 0.86 (0.81, 0.91) | 0.81 (0.76, 0.86) |
|  | 100 | 0.89 (0.82, 0.96) | 0.80 (0.74, 0.87) |
